# Assessment of Essential and Toxic Elements in Commercial Diet Catering Meal Plants in Poland: Compliance with Nutritional Recommendations

**DOI:** 10.3390/foods15142459

**Published:** 2026-07-11

**Authors:** Dominika Patrycja Dobiecka, Monika Grabia-Lis, Justyna Moskwa, Martyna Falkowska, Katarzyna Socha, Sylwia Katarzyna Naliwajko

**Affiliations:** Department of Bromatology, Faculty of Pharmacy with the Division of Laboratory Medicine, Medical University of Białystok, 15-222 Białystok, Poland; monika.grabia@umb.edu.pl (M.G.-L.);

**Keywords:** nutrients, daily food rations, trace elements, selected minerals, toxic elements, safety, toxicity, sustainable food systems

## Abstract

Background/Objectives: Commercial meal delivery diets are increasingly used as a convenient alternative to home-prepared meals. However, limited data are available regarding their mineral composition and potential exposure to toxic elements. This study aimed to evaluate the content of selected essential minerals (Ca, Cu, Fe, Mg, Se and Zn) and toxic elements (Cd and Pb) in daily food rations (DFRs) offered by selected commercial catering services in Poland. Methods: DFRs representing three dietary models (Hashimoto, DASH, and low-carb diets) were collected from commercial catering providers. Concentrations of essential minerals were determined using atomic absorption spectrometry (AAS), whereas toxic elements were determined using inductively coupled plasma mass spectrometry (ICP-MS). Mineral adequacy was assessed using Estimated Average Requirement (EAR) and Tolerable Upper Intake Level (UL) reference values. Exposure to toxic elements was evaluated using Estimated Daily Intake (EDI), estimated weekly Intake (EWI), Target Hazard Quotient (THQ), and Carcinogenic Risk (CR) indices. Results: Ca was the nutrient most frequently supplied in insufficient amounts, with 80–98% of analyzed meal plans failing to meet the EAR. In contrast, the remaining minerals were generally supplied in adequate amounts. Nevertheless, excessive intake of selected minerals was observed in some dietary models, with up to 37% of DASH diets exceeding the UL for Zn and approximately 32% of Hashimoto diets exceeding the UL for Cu. Although Cd and Pb were detected in all analyzed DFRs, THQ and CR values indicated negligible health risk. Conclusions: The analyzed meal delivery diets generally provided adequate amounts of most investigated minerals and did not pose a significant health risk related to Cd or Pb exposure. However, these findings apply only to the analyzed meal plans and should not be generalized to all commercial catering services or seasonal menu cycles in Poland. The widespread inadequacy of Ca intake and the occurrence of excessive Zn and Cu intake in selected dietary models highlight the need for improved nutritional quality control of commercially prepared diets.

## 1. Introduction

Over recent years, a dynamic increase in interest in dietary catering services has been observed [1,2]. These services constitute a convenient alternative to traditional meal preparation, particularly among individuals leading intense lifestyles and those concerned with health and body composition [3]. However, with the growing popularity of such solutions, there is an increasing need for a reliable assessment of their nutritional value and health safety [4].

One of the key aspects of diet quality is the content of mineral components, both essential for proper body functioning and potentially toxic. Elements such as Ca, Cu, Fe, Mg, Se, and Zn play an important role in numerous metabolic processes, including the functioning of the nervous and immune systems, as well as in maintaining overall homeostasis. Deficiencies of these elements may lead to serious health disorders [5,6,7]. On the other hand, the presence of toxic elements such as Cd and Pb, even in small amounts, may pose a risk to human health due to their cumulative and toxic properties [8,9,10].

Another important issue is the bioavailability of elements present in food, which depends not only on their total content but also on their chemical form, interactions between dietary components, and methods of food processing and preparation [11,12]. In the case of meal delivery diets, where meals undergo technological processing, transport, and storage, these factors may significantly affect the final nutritional value [13]. Therefore, the declared nutrient content may not accurately reflect its actual bioavailability to the body.

Additionally, in the context of increasing globalization of the food market and the introduction of new raw materials and production technologies, monitoring the presence of environmental contaminants in food is becoming increasingly important [14]. Toxic elements may originate from both plant and animal raw materials, as well as from production processes and packaging [15,16]. Consequently, systematic control of toxic elements content in ready-to-eat food products, including meal delivery diets, constitutes an essential element of ensuring consumer safety [17].

In the context of meal delivery diets, it is therefore particularly important to determine whether they provide adequate amounts of essential macro- and micronutrients, while at the same time not exceeding permissible levels of harmful elements. Despite the growing availability of such services on the market, data on their mineral composition and compliance with current nutritional standards remain limited.

The aim of this study was to assess the content of selected elements of nutritional and toxicological relevance in commercially available meal delivery diets. The obtained results were compared with current dietary recommendations and applicable safety standards in order to determine whether the analyzed menus meet the requirements for food quality and safety.

## 2. Materials and Methods

### 2.1. Materials

The study included 120 Daily Food Rations (DFRs) obtained from commercial dietary catering services operating in Poland. Sampling began in November 2022 and was completed in January 2024. A total of 40 catering providers located in Białystok were selected based on service availability and access to detailed menu information. Although meals were collected in Białystok, many providers operate nationwide and offer delivery across Poland. The sampling strategy was non-probabilistic; therefore, the results cannot be considered representative of the entire Polish dietary catering market. The selection aimed to include commonly used services while maintaining standardized sampling conditions. From each provider, three DFRs were collected over three consecutive days. Orders were placed anonymously via the official websites or mobile applications, following a standard consumer purchasing procedure. The use of consecutive days reduced short-term variability; however, weekly variation was not captured. Furthermore, sampling was performed only during the autumn–winter months, which limits the ability to evaluate seasonal differences throughout the year. Menu selection followed the natural rotation offered by each provider and was not randomized. Sampling was conducted on the same consecutive weekdays (e.g., Monday–Wednesday) for all providers to ensure consistency. Only meal plans with a declared energy value of 2000 kcal per day were included. This standardization enabled a direct comparison between samples and minimized variability related to caloric differences. The 2000 kcal level was used as a reference intake for an average adult, consistent with dietary guidelines. The selected providers represent commonly used and widely available catering services in Poland, as confirmed by public rankings and comparison platforms. Therefore, the included companies reflect a relevant segment of the commercial boxed diet market. The analysis covered three types of dietary plans: diets for individuals with Hashimoto’s disease (*n* = 45), the DASH diet (*n* = 45), and low-carbohydrate diets (*n* = 30). These diets were selected due to their popularity and classification as health-oriented or condition-specific meal plans, which are expected to comply with dietary recommendations. Depending on the diet type, DFRs consisted of three meals per day (low-carbohydrate diets) or five meals per day (Hashimoto and DASH diets), with a mean energy value of 2000 (±79.43) kcal. The number of meals differed between diet types but was not analyzed as an independent variable due to complete confounding with diet category. Meal plans typically included breakfast, lunch, dinner, and additional snacks (e.g., a second breakfast and an afternoon snack), in accordance with the structure declared by the providers. Information on meal composition, including ingredients, preparation methods, and added condiments, was obtained from online menus or product descriptions. All analyses were performed on meals as delivered, without modification of portion sizes, to reflect actual consumer exposure. Total food mass and portion sizes were not standardized, reflecting variability between providers. Declared nutritional values (energy and macronutrients) were recorded when available to verify compliance with the 2000 kcal standard. These data were not independently validated beyond comparison with delivered meals; therefore, discrepancies between declared and actual composition cannot be excluded.

### 2.2. Microwave Digestion of Samples

All samples were homogenized using a stainless-steel mill prior to analysis. Representative portions (0.2–0.3 g) were accurately weighed and transferred into polytetrafluoroethylene (PTFE) digestion vessels. Subsequently, 4 mL of spectrally pure concentrated nitric acid (69% HNO_3_; Tracepur, Merck, Darmstadt, Germany) was added. The acid digestion procedure was performed according to the manufacturer’s recommended protocol for the microwave digestion system. Microwave-assisted digestion was performed in a closed-vessel system (Berghof Speedwave, Eningen, Germany) under controlled temperature and pressure conditions. The digestion procedure consisted of four sequential stages: 10 min at 170 °C and 20 atm (80% power), followed by 10 min at 190 °C and 30 atm (90% power), then 10 min at 210 °C and 40 atm (90% power), and a final cooling step of 18 min to 50 °C at 40 atm with the microwave power switched off. After complete mineralization, the digests were quantitatively transferred to polypropylene tubes and diluted to the appropriate volume with deionized water. The resulting digests were diluted depending on the element being determined: Fe and Zn—3-fold, Se—2-fold, Ca and Cu—10-fold, Mg—25-fold, and Cd and Pb—20-fold (excluding the dilution resulting from the digestion process itself). The samples were not filtered after dilution because the digests were clear and free of visible particulates. After dilution, the solutions were thoroughly vortex-mixed before analysis. Certified reference materials and working blanks were prepared and processed following the same digestion and dilution procedure as the analyzed samples. The concentrations of the analyzed elements, including macroelements, essential trace elements, and toxic elements, were determined and expressed as mg/kg or µg/kg of the product.

### 2.3. Determination of Mineral Components

#### 2.3.1. Ca, Fe, Mg, Zn

The concentrations of Ca, Fe, Mg and Zn were determined by flame atomic absorption spectrometry (FAAS) using a Z-2000 Tandem Flame/Furnace AA Spectrophotometer (Hitachi, Tokyo, Japan) with flame atomization (acetylene–air). The analytical wavelengths were set at 422.7 nm for Ca, 248.3 nm for Fe, 285.2 nm for Mg, and 213.9 nm for Zn, applying Zeeman background correction.

For Ca and Mg determination, a 1% lanthanum (III) chloride hydrate solution (Sigma-Aldrich, St. Louis, MO, USA) was used as a phosphate-masking agent. The limits of detection (LOD) were 0.12 mg/kg for Ca, 0.14 mg/kg for Fe, 0.008 mg/kg for Mg, and 0.019 mg/kg for Zn.

#### 2.3.2. Se, Cu

The concentrations of Se and Cu were determined by electrothermal atomic absorption spectrometry (ETAAS) with graphite furnace atomization using the same instrument (Z-2000, Hitachi, Tokyo, Japan). For Se analysis, a palladium–magnesium matrix modifier (Sigma-Aldrich, Merck, Darmstadt, Germany) was applied to enhance signal stability and accuracy. Cu was measured under optimized furnace conditions without the use of a chemical modifier. All measurements were performed with Zeeman background correction. The LOD values were 1.11 μg/kg for Se and 0.55 μg/kg for Cu.

#### 2.3.3. Cd, Pb

The determination of Cd and Pb was carried out using inductively coupled plasma mass spectrometry (ICP-MS) with a NexION 300D ICP-MS (PerkinElmer, Waltham, MA, USA), operated in standard mode. Measurements were performed at atomic masses of 109.903, 110.904, 112.905, and 113.904 for Cd, and 205.975, 206.976, and 207.977 for Pb.

To limit polyatomic interferences, collision-based processes were applied. Each sample was analyzed in five replicates using dual detector calibration. The dwell time per atomic mass unit was set to 50 ms, and the total integration time was 1000 ms. The LOD values were 0.052 μg/kg for Cd and 0.37 μg/kg for Pb.

#### 2.3.4. Accuracy Check of the Methods

The certified reference materials (CRMs) were used to control the quality of the performed analyses (Table 1). Simulated Diet D (Livsmedelsverket, National Food Agency, Uppsala, Sweden) was used for all analyzed elements except Se, for which mushroom powder (CS–M-3) was used (Institute of Nuclear Chemistry and Technology, Warsaw, Poland).

### 2.4. DFRs as Sources of Dietary Minerals

The adequacy of mineral intake provided by the analyzed DFRs was assessed using Estimated Average Requirement (EAR) and Tolerable Upper Intake Level (UL). The analysis included Ca, Cu, Fe, Mg, Se and Zn. Reference EAR values for adults were adopted according to current nutritional recommendations [18]. All calculations were based on the total daily food supply delivered within each catering plan. To ensure comparability between samples, analyses were standardized to a declared daily energy intake of 2000 kcal, which represents a commonly accepted reference intake for adults. Mineral content was determined for meals as delivered, without modification of portion size or composition. For each analyzed mineral, the percentage of EAR covered by the daily menu was calculated. Furthermore, the proportion of diets providing mineral amounts below the EAR and exceeding the UL was determined for each dietary model. Intake values below the EAR were interpreted as potentially inadequate, whereas values above the UL indicated a negligible risk of excessive mineral intake. No UL analysis was performed for Mg, as the established UL applies only to magnesium from supplements, fortified foods, and readily dissociable magnesium salts, not to naturally occurring magnesium in foods.

### 2.5. Risk Assessment

Short-term exposure to toxic elements was evaluated using indicators such as estimated daily intake (EDI) and estimated weekly intake (EWI) [19]. In contrast, long-term exposure was assessed based on parameters including the target hazard quotient (THQ) [20] and cancer risk (CR) [21].

The level of oral exposure to toxic elements was quantified by calculating EDI values using the following equation:

EDI=C×Cons,

where C represents the mean concentration of a given toxic element in the analyzed meal delivery diets (mg/kg), and Cons denotes the average daily consumption of the diet (kg/d), corresponding to the total mass of the daily ration standardized to 2000 kcal. The calculations were based on the actual composition of the daily menus provided by catering services.

Based on the EDI values, the estimated weekly intake (EWI) was determined by multiplying the daily intake by a factor of 7, reflecting weekly exposure. For consistency with the THQ and CR calculations, the EDI values are expressed in mg/day and were converted from the daily intake values (μg/day), using the conversion factor of 1 mg = 1000 μg.

To evaluate non-carcinogenic risk associated with long-term exposure, the target hazard quotient (THQ) was calculated using the following equation:

$$THQ=\left(Fr\times D\times Cons\times C\right)/(RfD\times BW\times T)\times 10^{-3},$$

where Fr represents exposure frequency (365 days/year), D is the duration of exposure (70 years), Cons denotes the average daily consumption of the diet (g/day), C is the mean concentration of the analyzed toxic element, and RfD is the oral reference dose, BW is the assumed adult body weight (70 kg), and T is the averaging time for non-carcinogenic effects. In the EDI equation, Cons is expressed in kg/day to correspond with the concentration values (mg/kg), whereas in the THQ equation it is expressed in g/day according to the original US EPA methodology; therefore, the conversion factor of 10^−3^ is applied to convert grams to kilograms. The RfD values were adopted as 1 μg/kg BW/day for Cd and Pb, in accordance with the United States Environmental Protection Agency (US EPA) guidelines [22]. A THQ value greater than 1 suggests a potential non-carcinogenic risk, whereas values below 1 indicate negligible risk.

The carcinogenic risk (CR) was estimated to assess the probability of cancer development associated with exposure to toxic elements, using the following equation:

$$CR=\left(Fr\times D\times EDI\times Sf\right)/T\times 10^{-3},$$

where Sf is the carcinogenic slope factor. The slope factors applied in this study were 6.3 mg/kg/day for Cd, and 0.0085 mg/kg/day for Pb, as adopted from published risk assessment methodology [23,24,25]. Since carcinogenic risk assessment for Pb remains associated with considerable uncertainty due to the lack of a universally accepted safe exposure threshold and the absence of an officially established oral slope factor in the current US EPA IRIS database, the estimated CR values for Pb should be interpreted with caution. A CR value exceeding 10^−4^ is considered indicative of elevated carcinogenic risk [22].

### 2.6. Data Analyses

Data preprocessing and statistical analyses were performed using Python 3.12 (SciPy 1.17.1, NumPy 2.0.0, Pandas 2.2.2, Pillow 12.2.0).

Descriptive statistics included mean, standard deviation, median, Q1–Q3, minimum and maximum. Box plots were generated in Python using the Pillow library and embedded into Excel workbooks using openpyxl. For each dietary model, median, interquartile range, whiskers and outliers were calculated. Pairwise differences between diets were assessed using the Mann–Whitney U test with Holm correction for multiple comparisons. Significance levels were marked as * *p* < 0.05, ** *p* < 0.01, *** *p* < 0.001, and ns for non-significant results. Macroelement intake (Ca, Mg) and microelement intake (Cu, Fe, Se, Zn) were compared between Hashimoto, DASH, and low-carb diets. For nutritional adequacy, the percentage of diets meeting EAR, exceeding UL, and falling below the recommended intake was calculated. Toxic elements were assessed using EDI, EWI, THQ and CR values. Cancer risk (CR) for Cd and Pb was presented on a logarithmic scale, with the acceptable risk range marked between 1 × 10^−6^ and 1 × 10^−4^.

## 3. Results

### 3.1. Ca, Cu, Fe, Mg, Se, Zn

**Figure 1:**
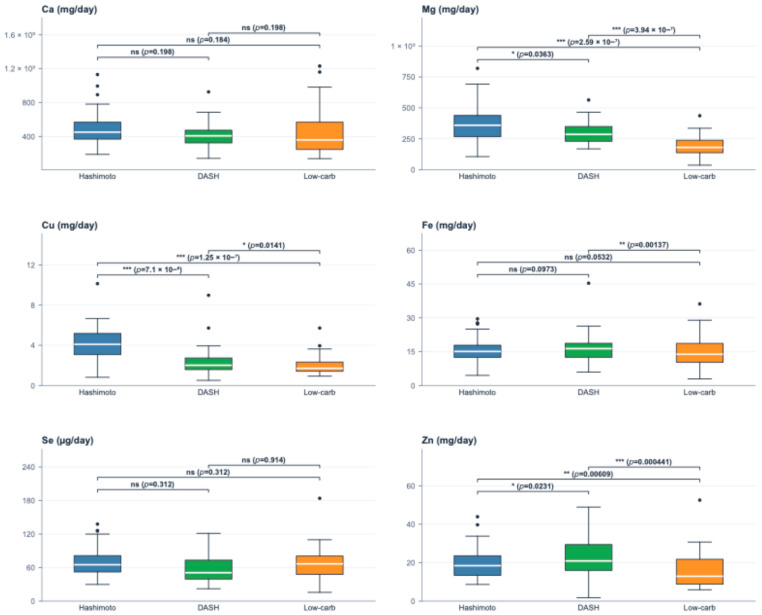
Distribution of estimated daily intake of selected mineral elements in DFRs across Hashimoto, DASH and low-carb diets. Box plots show median, interquartile range and variability for Ca, Cu, Fe, Mg, Se and Zn. Significant differences between diet groups are marked with asterisks: * *p* < 0.05, ** *p* < 0.01, and *** *p* < 0.001; ns indicated non-significant differences.

**Figure 2:**
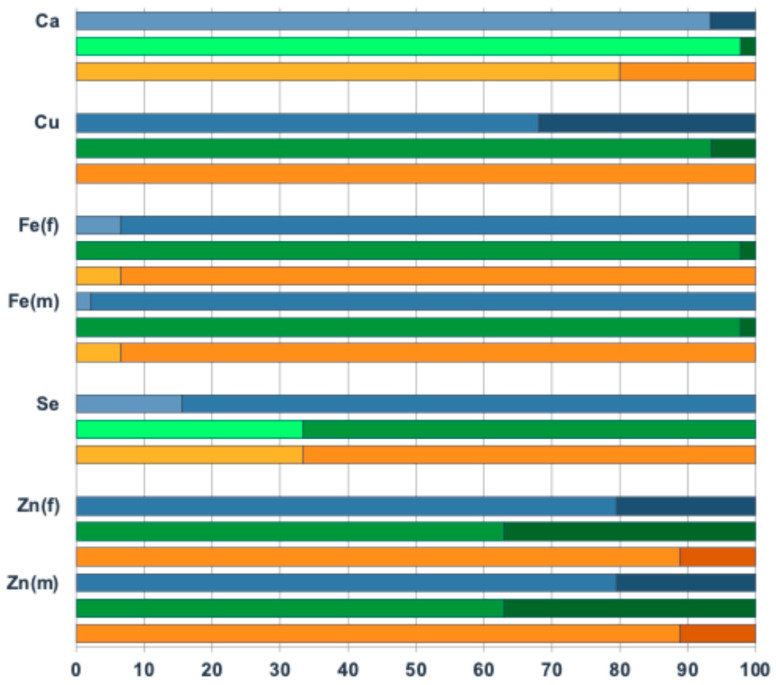
Distribution of daily food rations according to mineral intake adequacy across diet types. Horizontal stacked bars show the percentage of daily food rations classified as below the Estimated Average Requirement (EAR), within the adequate range between EAR and UL, or above the Tolerable Upper Intake Level (UL) for selected minerals. For each mineral, three bars represent the analyzed diet types: Hashimoto shown in blue shades, DASH in green shades, and low-carb in orange shades. Lighter segments indicate values below EAR, medium shades indicate values within the reference range, and darker segments indicate values exceeding UL. Sex-specific reference values are indicated as f for female and m for male standards.

Detailed data on mineral adequacy expressed as EAR coverage (%) according to diet type are presented in Table 2, while detailed mineral composition data are presented in Table 3 and Figure 1. The proportions of food rations with mineral content below the EAR, within the recommended range, and above the UL are presented in Figure 2.

The analysis of mineral intake revealed notable differences between the evaluated dietary models. Regarding Ca, the highest mean intake was observed in the Hashimoto diet group (488.1 ± 193.8 mg/day), whereas the lowest intake was recorded in the DASH group (303.4 ± 83.7 mg/day). In the low-carb diet group, the mean Ca intake amounted to 449.3 ± 295.5 mg/day. Despite the relatively higher Ca intake in the Hashimoto and low-carb diets, the average intake in all analyzed groups remained substantially below the EAR for the Polish population, established at 800 mg/day. Moreover, a very high proportion of dietary models failed to meet the EAR for Ca, including 93.33% in the Hashimoto group, 97.78% in the DASH group and 80.00% in the low-carb group. These findings may indicate an increased risk of inadequate calcium intake associated with the analyzed dietary models. At the same time, all observed values remained markedly below the tolerable UL of 2500 mg/day, and no diets exceeded the UL threshold, suggesting no risk of excessive calcium intake.

Cu intake exceeded the EAR value of 0.7 mg/day in all dietary groups. The highest mean Cu intake was identified in the Hashimoto diet group (4.1 ± 1.8 mg/day), followed by the DASH diet (2.5 ± 1.5 mg/day), while the lowest intake was observed in the low-carb group (1.9 ± 0.9 mg/day). Only a very small percentage of diets failed to meet the EAR for Cu (4.44%), indicating an overall adequate dietary supply of this mineral. Mean Cu intake in the Hashimoto group approached the UL value of 5 mg/day, and the maximum recorded intake (10.13 mg/day) exceeded this threshold. Nevertheless, the proportion of diets exceeding the UL for Cu remained relatively low, accounting for 31.82% in the Hashimoto group and 6.52% in the DASH group, while no exceedances were observed in the low-carb diet. These findings suggest that excessive copper intake may occur in selected dietary models, particularly in the Hashimoto diet.

For Mg, the highest mean intake was observed in the Hashimoto diet group (366.7 ± 152.0 mg/day), reaching values close to the recommended intake for men (330–350 mg/day) and exceeding the requirements for women (255–265 mg/day). The DASH diet was characterized by a moderate Mg intake (303.4 ± 83.7 mg/day), whereas the lowest intake was identified in the low-carb group (189.8 ± 77.1 mg/day). These findings suggest that low-carb dietary models may be particularly susceptible to insufficient magnesium intake.

Analysis of Fe intake demonstrated that mean values in all groups exceeded the EAR for adult men and women (6–8 mg/day). The highest Fe intake was reported in the DASH group (17.7 ± 5.7 mg/day), followed by the Hashimoto diet group (15.8 ± 5.5 mg/day), whereas the lowest intake was observed in the low-carb diet group (13.9 ± 7.2 mg/day). Only a small percentage of diets had Fe content below the EAR, ranging from 0% to 6.67% depending on sex and dietary group, indicating generally adequate iron intake across the analyzed dietary models. Moreover, mean Fe intake in all dietary groups remained below the UL value of 40 mg/day. Exceeding the UL for Fe was rare and observed only in the DASH group (2.17%).

Se intake exceeded the EAR value of 45 μg/day in all analyzed dietary groups. The highest mean intake was observed in the Hashimoto diet group (69.4 ± 25.8 μg/day), while the DASH and low-carb diets demonstrated comparable values of 60.1 ± 23.6 μg/day and 61.4 ± 32.6 μg/day, respectively. Nevertheless, 15.56% of diets in the Hashimoto group and 33.33% in both the DASH and low-carb groups did not achieve the EAR for Se. Despite this, all mean Se intakes remained substantially below the UL of 255 μg/day, and no diets exceeded the UL threshold, indicating a low risk of Se overconsumption.

With regard to Zn, the highest mean intake was reported in the DASH group 23.1 ± 9.5 mg/day, followed by the Hashimoto diet group (19.5 ± 7.8 mg/day), whereas the lowest intake was observed in the low-carb group (15.2 ± 10.1 mg/day). All recorded values substantially exceeded the EAR range established for the Polish population (6.8–9.4 mg/day), indicating an overall adequate dietary supply of Zn in all analyzed groups. However, inadequate Zn intake was still observed in a subset of diets, particularly among men following the low-carb diet, where 40.00% did not meet the EAR value. Additionally, the mean Zn intake in the DASH group approached the UL value of 25 mg/day, while the percentage of diets exceeding the UL ranged from 11.11% in the low-carb group to 36.96% in the DASH group, suggesting a potential risk of excessive Zn intake in selected dietary models.

### 3.2. Cd, Pb

Levels of toxic elements determined in the DFRs are presented in Table 4. The mean Cd concentration across all analyzed samples was 84.74 ± 79.47 μg/d. The highest Cd levels were observed in the Hashimoto diet group (110.45 ± 90.60 μg/day), whereas the lowest levels were recorded in the low-carb diet group (42.71 ± 20.34 μg/day). The mean Pb concentration across all analyzed samples was 161.45 ± 136.92 μg/day. The highest Pb levels were detected in the low-carb diet group (237.25 ± 181.29 μg/day), while the lowest concentrations were observed in the Hashimoto diet group (77.79 ± 50.49 μg/day).

Health risk indices for the individual dietary patterns were calculated according to the equations described in the Materials and Methods section. Table 5 summarizes the EDI and EWI (calculated as EDI × 7). For all analyzed diets, the mean EDI for Cd was 0.085 mg/day. The highest values were observed for the Hashimoto diet (0.110 mg/day), whereas the lowest values were recorded for the low-carb diet (0.043 mg/day). In the case of Pb, the mean EDI across all diets was 0.161 mg/day. Group-specific analysis demonstrated the highest values in the low-carb diet (0.237 mg/day) and the lowest in the Hashimoto diet (0.078 mg/day).

No elevated health risk associated with the intake of the investigated toxic elements through the analyzed diets was identified, as all calculated values remained below the reference threshold of 1. The THQ and CR indices are presented in Table 6. The mean THQ value for Cd across all samples was 0.0012 ± 0.0011. Among the analyzed dietary groups, the highest THQ value was observed for the Hashimoto diet (0.0016 ± 0.0011), while the lowest was noted for the low-carb diet (0.0006 ± 0.0003). The mean THQ value for Pb was 0.0023 ± 0.0020. The highest THQ level for Pb was identified in the low-carb diet group (0.0034 ± 0.0026), whereas the lowest values were recorded for the Hashimoto diet group (0.0011 ± 0.0007).

**Figure 3:**
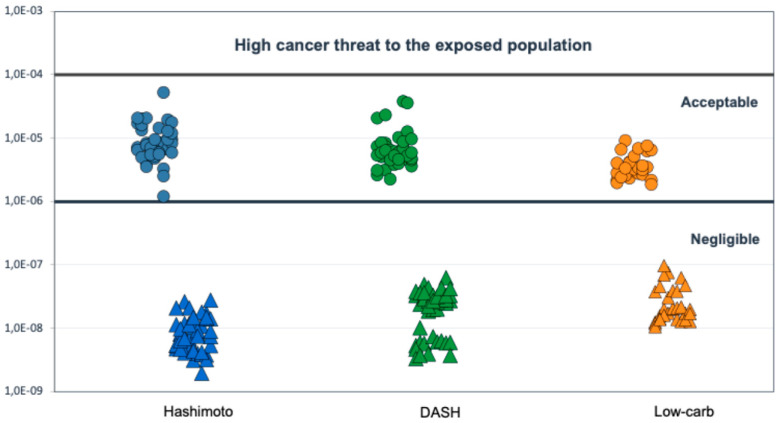
Estimated cancer risk associated with dietary exposure to lead (Pb) and cadmium (Cd) across diet types. The scatter plot presents individual risk estimates on a logarithmic scale for three diet types. Color coding as in Figure 2. Circles represent Pb, while triangles represent Cd. Horizontal reference lines indicate commonly used risk interpretation thresholds: values below 1 × 10^−6^ are considered negligible or insignificant, values between 1 × 10^−6^ and 1 × 10^−4^ fall within the acceptable or tolerable risk range, and values above 1 × 10^−4^ indicate a high cancer threat to the exposed population. In the analyzed samples, Pb-related risk values were generally within the acceptable/tolerable range, whereas Cd-related risk values remained below the negligible-risk threshold.

The carcinogenic risk coefficient (CR) for elements with confirmed carcinogenic potential was estimated based on the obtained analytical results and available statistical data for the Polish population. The results indicate that the risk of cancer development associated with the consumption of the analyzed diets is low (Figure 3).

## 4. Discussion

The present study evaluated the mineral composition and selected toxic elements concentrations in commercially available meal delivery diets, including Hashimoto, DASH, and low-carb dietary models. The results indicate substantial differences in the supply of essential minerals between diet types, while simultaneously demonstrating a generally low health risk associated with exposure to Cd and Pb through the analyzed dietary patterns.

Among the analyzed minerals, Ca appeared to be the most critical nutrient of concern. Between 80% and 98% of the analyzed meal plans failed to meet the EAR, depending on dietary type. Even in the Hashimoto and low-carb diets, which provided higher Ca levels than the DASH diet, average Ca intake remained below the recommended 800 mg/day. Similar observations have been reported in previous assessments of commercial catering diets and self-selected diets, where Ca is frequently identified as one of the nutrients most susceptible to inadequate intake [26,27]. Ca plays a fundamental role in skeletal health, muscle contraction, nerve transmission, and intracellular signaling [28]. Therefore, long-term consumption of diets characterized by insufficient Ca content may contribute to reduced bone mineral density and increased risk of osteoporosis, particularly among women and older adults. The widespread inadequacy observed in the present study suggests that meal delivery services should place greater emphasis on incorporating calcium-rich foods, such as dairy products, fortified plant-based alternatives, and calcium-containing mineral waters. Although no ingredient-level analysis was performed, a review of the available menu descriptions suggests that the low Ca content may be related to the relatively limited inclusion of dairy products and calcium-fortified foods in the analyzed meal plans. This pattern was observed across all three dietary models rather than being restricted to a single diet type, indicating that insufficient incorporation of calcium-rich ingredients may represent a broader feature of the analyzed menus. Nevertheless, this interpretation should be considered with caution, as differences between individual catering providers and recipes were not evaluated.

In contrast to Ca, Fe intake was generally adequate across all dietary models. This finding is consistent with the presence of foods recognized as major sources of dietary Fe, including meat, fish, legumes, and fortified products, which are important contributors to Fe intake in mixed diets and support adequate Fe status [29,30]. The low prevalence of diets failing to meet Fe requirements suggests that the analyzed catering services were successful in maintaining an appropriate supply of this nutrient.

The observed differences in Mg intake between dietary models deserve particular attention. While the Hashimoto diet provided Mg amounts close to recommended values, the low-carb diet supplied considerably lower levels, suggesting a greater risk of inadequate intake among individuals regularly consuming this dietary pattern. The lower Mg supply in low-carb diets is likely associated with reduced consumption of whole grains, legumes, and certain fruits, which represent major dietary sources of this mineral [31]. Since Mg is involved in numerous physiological processes, including energy metabolism and cardiovascular regulation, inadequate intake over extended periods may have adverse health consequences [32]. These findings emphasize the importance of careful essential minerals planning when developing carbohydrate-restricted meal plans.

Se intake was generally satisfactory; however, approximately one-third of DASH and low-carb meal plans failed to meet the EAR, indicating that inadequate Se supply may still occur despite satisfactory mean intake values. Adequate Se intake is often considered particularly important in dietary strategies targeted at individuals with Hashimoto’s thyroiditis [33]. The variability observed among meal plans suggests that the inclusion of selenium-rich foods may not be sufficiently standardized across catering services.

The results also indicate that Zn and Cu intake were generally sufficient; however, excessive intake was observed in a proportion of diets. In particular, up to 37% of DASH diets exceeded the UL for Zn, while approximately one-third of Hashimoto diets exceeded the UL for Cu. Although occasional exceedances are unlikely to result in adverse effects, chronic excessive intake may disturb mineral homeostasis and contribute to nutrient interactions, particularly between Zn and Cu [34,35]. These findings highlight that nutritional adequacy should be assessed not only in terms of deficiency prevention but also with respect to the avoidance of excessive essential minerals intake. Based on the available menu descriptions, the elevated Zn and Cu levels may reflect the frequent inclusion of foods naturally rich in these minerals, such as meat, nuts, seeds, and whole-grain products. However, because the present study evaluated complete daily food rations rather than individual ingredients, it was not possible to identify the specific meal components responsible for the observed exceedances.

In addition to nutritional quality, the present study assessed the safety of meal delivery diets with regard to exposure to toxic elements. Cd and Pb were detected in all analyzed dietary models, which is consistent with the ubiquitous presence of these contaminants in the food chain. Nevertheless, all calculated THQ values remained substantially below the critical threshold of 1, indicating negligible non-carcinogenic risk. Similarly, the estimated carcinogenic risk associated with the analyzed diets was low. These findings suggest that, despite measurable contamination levels, the evaluated meal plans do not constitute a significant source of Cd or Pb exposure for consumers.

Differences in contaminant concentrations between dietary models may be attributable to variations in ingredient composition. Cd exposure is frequently associated with cereals, vegetables, and plant-derived products, whereas Pb contamination may originate from a broad range of environmental sources affecting both plant- and animal-based foods [36,37,38,39]. However, the observed differences did not translate into elevated health risk indicators, supporting the overall safety of the investigated dietary models in terms of exposure to the analyzed toxic elements.

The present findings have practical implications for both consumers and meal delivery providers. Catering diets are often marketed as nutritionally balanced solutions that facilitate healthy eating habits. However, the results demonstrate that compliance with general dietary recommendations does not necessarily ensure adequate essential minerals supply. In particular, the consistently low Ca content observed across all dietary models indicates a need for improved nutritional quality control and menu optimization.

### Study Limitations

Several limitations should be taken into consideration when interpreting the findings of the present study. First, the analysis was based on DFRs obtained from dietary catering companies commercially available on the Polish market at the time of the study. Therefore, while the findings provide a valuable overview of the nutritional quality and safety of the analyzed commercially available dietary catering meal plans, they may not be fully generalizable to meal delivery markets in other countries. In addition, the study focused on three specific dietary models, namely Hashimoto, DASH, and low-carb diets, which may limit the applicability of the findings to other types of catering diets available to consumers. Another limitation concerns the sampling strategy; DFRs from each provider were collected over a period of three consecutive days, which may not adequately capture potential variations in menu composition occurring over longer time periods, including weekly menu cycles or seasonal changes in ingredient availability. Furthermore, since three DFRs were collected from each provider, observations within the same provider may not be fully independent, and the reported *p*-values should therefore be interpreted with appropriate caution. Potential differences between individual catering companies were not analyzed separately for this reason. Although the assessment was performed at the level of complete DFRs, providing a realistic estimate of total daily nutrient and contaminant exposure, it did not allow identification of specific meals or food products contributing most to mineral intake or toxic elements exposure. Additionally, only total concentrations of minerals and toxic elements were determined. Factors influencing nutrient bioavailability, as well as potential interactions among dietary components affecting mineral absorption and utilization, were not evaluated. Future studies should therefore include a broader range of catering services, longer sampling periods, seasonal assessments, and analyses of nutrient bioavailability to provide a more comprehensive evaluation of the nutritional quality and safety of meal delivery diets.

These findings indicate that while meal delivery diets can represent a safe and convenient dietary option, further optimization of their mineral composition is warranted to improve overall nutritional quality.

## 5. Conclusions

The present study indicates that analyzed commercially available dietary catering meal plans may constitute a convenient dietary option, as the analyzed DFRs generally provided adequate amounts of most minerals and were associated with low exposure to Cd and Pb. Nevertheless, the consistently inadequate Ca content observed across all dietary models highlights an important nutritional limitation of these products. The occurrence of excessive Zn and Cu intake in selected meal plans further emphasizes the need for comprehensive essential minerals monitoring during menu development. Regular nutritional evaluation of catering diets should therefore include both nutrient adequacy and food safety assessments to ensure their long-term health benefits for consumers.

## Figures and Tables

**Table 1:** The results obtained in the method validation.

Element	Detection Limit for Method	Recovery for CRM	Precision (%)
**Ca**	0.12 mg/kg	99.4	3.0
**Cd**	0.052 μg/kg	101.1	2.2
**Cu**	0.55 μg/kg	98.9	2.2
**Fe**	0.14 mg/kg	99.2	3.9
**Mg**	0.008 mg/kg	98.5	4.5
**Pb**	0.37 μg/kg	98.6	4.4
**Se**	1.11 μg/kg	100.7	2.8
**Zn**	0.019 mg/kg	101.2	3.9

**Table 2:** Dietary mineral adequacy expressed as EAR coverage (%) according to diet type.

Type of Diets	EAR (%)					
	Ca	Cu	Fe	Mg	Se	Zn
	Male/Female					
Hashimoto	61	590	263/197	144 (138)/111 (105)	154	286/207
DASH	38	357	295/221	119 (115)/92 (87)	133	339/245
Low-carb	56	267	231/173	74 (72)/58 (54)	137	222/161
Total	57	422	267/200	117 (113)/91 (85)	142	290/210

EAR (Estimated Average Requirement); Ca: 800 mg, Cu: 0.7 mg, Fe: 6–8 mg, Mg: 255 (265)–330 (350) mg, Se: 45 μg, Zn: 6.8–9.4 mg.

**Table 3:** The content of studied elements (Ca, Cu, Fe, Mg, Se, Zn) measured in DFRs.

Type of Diets	Ca [mg/day]		Cu [mg/day]	
	Av. ± SD Min–Max	Me Q_1_–Q_3_	Av. ± SD Min–Max	Me Q_1_–Q_3_
Hashimoto	488.1 ± 193.8 188.2–1130.6	451.1 368.9–567.9	4.1 ± 1.8 0.8–10.1	4.1 3.1–5.2
DASH	303.4 ± 83.7 168.4–563.4	297.9 253.4–349.4	2.5 ± 1.5 0.5–9.0	2.3 1.7–3.0
Low-carb	449.3 ± 295.5 138.4–1230.6	340.3 244.8–609.8	1.9 ± 0.9 0.8–4.0	1.4 1.4–2.1
Total	456.5 ± 212.1 138.4–1230.6	408.8 317.9–564.4	2.9 ± 1.8 0.5–10.1	2.4 1.7–2.3
	**Fe [mg/day]**		**Mg [mg/day]**	
	**Av. ± SD** **Min–Max**	**Me** **Q_1_–Q_3_**	**Av. ± SD** **Min–Max**	**Me** **Q_1_–Q_3_**
Hashimoto	15.8 ± 5.5 4.5–29.5	15.1 12.4–17.9	366.7 ± 152.0 106.1–819.7	359.8 267.1–439.4
DASH	17.7 ± 5.7 10.0–45.4	16.9 14.4–19.8	303.4 ± 83.7 168.4–563.4	297.9 253.4–349.4
Low-carb	13.9 ± 7.2 2.9–36.2	12.1 9.0–15.1	189.8 ± 77.1 36.8–443.9	180.2 137.4–230.0
Total	16.0 ± 6.2 2.9–45.4	15.2 12.3–18.5	298.7 ± 131.6 36.8–819.7	280.4 206.5–370.5
	**Se [μg/day]**		**Zn [mg/day]**	
	**Av. ± SD** **Min–Max**	**Me** **Q_1_–Q_3_**	**Av. ± SD** **Min–Max**	**Me** **Q_1_–Q_3_**
Hashimoto	69.4 ± 25.8 29.8–137.8	65.1 52.2–81.7	19.5 ± 7.8 8.7–43.9	18.4 13.42–22.17
DASH	60.1 ± 23.6 22.3–121.3	57.5 42.8–77.3	23.1 ± 9.5 1.8–48.9	21.9 17.2–21.9
Low-carb	61.4 ± 32.6 15.7–184.1	59.3 42.8–72.1	15.2 ± 10.1 5.3–52.6	12.1 8.4–17.7
Total	63.9 ± 27.0 15.7–184.1	63.3 44.3–80.5	19.8 ± 9.5 1.8–52.6	18.5 13.0–25.5

Av.—average; SD—standard deviation; Max—maximum; Me—median; Min—Minimum; Q_1_—lower quartile; Q_3_—upper quartile.

**Table 4:** The content of Cd and Pb measured in DFRs.

Type of Diets	Cd [μg/day]		Pb [μg/day]	
	Av. ± SD Min–Max	Me Q_1_–Q_3_	Av. ± SD Min–Max	Me Q_1_–Q_3_
Hashimoto	110.45 ± 90.60 13.09–572.41	78.00 61.22–142.08	77.79 ± 50.49 16.19–230.28	64.75 38.92–97.00
DASH	87.05 ± 81.94 24.86–417.30	61.56 50.89–80.91	194.56 ± 119.55 27.29–519.44	219.16 52.16–268.59
Low-carb	42.71 ± 20.34 20,41–100.36	35.68 29.57–46.14	237.25 ± 181.29 86.94–803.15	162.31 113.98–315.79
Total	84.74 ± 79.47 13.09–572.41	64.11 41.99–90.77	161.45 ± 136.92 16.19–803.15	119.10 54.83–228.42

Av.—average; SD—standard deviation; Max—maximum; Me—median; Min—Minimum; Q_1_—lower quartile; Q_3_—upper quartile.

**Table 5:** Mean values of EDI and EWI in the analyzed DFRs.

Type of Diets	EDI [mg/day]/EWI [mg/week] Av. ± SD Min–Max			
	Cd		Pb	
Hashimoto	0.110 ± 0.091 0.013–0.572	0.773 ± 0.634 0.092–4.007	0.078 ± 0.050 0.016–0.230	0.545 ± 0.353 0.113–1.612
DASH	0.087 ± 0.082 0.025–0.417	0.609 ± 0.574 0.174–2.921	0.195 ± 0.120 0.027–0.519	1.362 ± 0.837 0.191–3.636
Low-carb	0.043 ± 0.020 0.020–0.100	0.299 ± 0.142 0.143–0.703	0.237 ± 0.181 0.087–0.803	1.661 ± 1.269 0.609–5.622
Total	0.085 ± 0.079 0.013–0.572	0.593 ± 0.556 0.092–4.007	0.161 ± 0.137 0.016–0.803	1.130 ± 0.958 0.113–5.622

Av.—average; EDI—estimated daily intake; EWI—estimated weekly intake; SD—standard deviation; Max—maximum; Min—Minimum.

**Table 6:** THQ and CR in the analyzed DFRs.

Type of Diets	THQ Av. ± SD Min–Max	
	Cd	Pb
Hashimoto	0.0016 ± 0.0011 0.0013–0.0082	0.0011 ± 0.0007 0.0002–0.0033
DASH	0.0012 ± 0.0012 0.0004–0.0060	0.0028 ± 0.0017 0.0004–0.0074
Low-carb	0.0006 ± 0.0003 0.0003–0.0014	0.0034 ± 0.0026 0.0012–0.0115
Total	0.0012 ± 0.0011 0.0002–0.0082	0.0023 ± 0.0020 0.0002–0.0115
Mean CR of all	0.0000515	0.0000001

Av.—average; CR—cancer risk; SD—standard deviation; Max—maximum; Min—Minimum; THQ—target hazard quotient.

## Data Availability

The raw data supporting the conclusions of this article will be made available by the authors on request.
